# Graves disease-induced thrombotic thrombocytopenic purpura: a case report 

**DOI:** 10.1186/s13256-019-2307-1

**Published:** 2019-12-13

**Authors:** Saira Chaughtai, Ijaz Khan, Varsha Gupta, Zeeshan Chaughtai, Raquel Ong, Arif Asif, Mohammad A. Hossain

**Affiliations:** Internal Medicine Residency Program, Department of Medicine, Hackensack Meridian School of Medicine at Seton Hall University, Jersey Shore University Medical Center, Hackensack Meridian Health, Neptune, NJ 07753 USA

**Keywords:** Graves disease, Thrombotic thrombocytopenic purpura

## Abstract

**Background:**

Thrombotic thrombocytopenic purpura is an autoimmune disease that carries a high mortality. Very few case reports in the literature have described a relationship between Graves disease and thrombotic thrombocytopenic purpura. We present a case of a patient with Graves disease who was found to be biochemically and clinically hyperthyroid with concurrent thrombotic thrombocytopenic purpura.

**Case presentation:**

Our patient was a 30-year-old African American woman with a history of hypertension and a family history of Graves disease who had recently been diagnosed with hyperthyroidism and placed on methimazole. She presented to our hospital with the complaints of progressive shortness of breath and dizziness. Her vital signs were stable. On further evaluation, she was diagnosed with thrombotic thrombocytopenic purpura, depending on clinical and laboratory results, and also was found to have highly elevated free T4 and suppressed thyroid-stimulating hormone. She received multiple sessions of plasmapheresis and ultimately had a total thyroidectomy. The patient’s hospital course was complicated by pneumonia and acute respiratory distress syndrome. Her platelets stabilized at approximately 50,000/μl, and her ADAMTS13 activity normalized despite multiple complications. The patient ultimately had a cardiac arrest with pulseless electrical activity and died despite multiple attempts at cardiopulmonary resuscitation.

**Conclusion:**

Graves disease is an uncommon trigger for the development of thrombotic thrombocytopenic purpura, and very few cases have been reported thus far. Therefore, clinicians should be aware of this association in the appropriate clinical context to comprehensively monitor hyperthyroid patients during treatment.

## Introduction

Autoimmune diseases tend to cluster not only in families but also in the same individual. Graves disease is an example of an autoimmune disease in which autoantibodies bind with hormone receptors of the thyroid gland, causing the thyroid to overproduce thyroid hormone [1]. It is characterized clinically by diffuse goiter, ophthalmopathy, hyperthermia, agitation, weight loss, palpitations, and even heart failure [1]. Thrombotic thrombocytopenic purpura (TTP) is an autoimmune disease caused by a deficiency of ADAMTS13, a von Willebrand Factor (vWF)-cleaving metalloprotease [2]. This leads to the buildup of vWF multimers, activating a cascade in the bloodstream leading to destruction of platelets by the large multimers. TTP is characterized by thrombocytopenia, hemolytic anemia, and evidence of end-organ damage. The treatment primarily is plasmapheresis to remove the autoantibodies from the bloodstream [2]. TTP carries a high mortality. Graves disease has been associated with immune thrombocytopenic purpura (ITP), but only a few case reports have described a possible association of Graves disease with TTP. We present a case in which a patient with Graves disease was found to be biochemically and clinically hyperthyroid with concurrent TTP.

## Case presentation

A 30-year-old African American woman with a history of hypertension, recently diagnosed with hyperthyroidism and receiving methimazole, presented to our hospital with the complaints of shortness of breath, dizziness, and weakness that had progressed over 2 days. She had no history of rash, bleeding from any sites of the body, diarrhea, or vomiting.

Her vital signs showed a blood pressure of 126/96 mmHg, pulse of 109 beats/minute, respirations of 16/minute, temperature of 98.2 °F, and pulse oxygenation of 100% on room air. On physical examination, she was obese, awake and alert, had marked conjunctival pallor, and had a diffuse and enlarged thyroid gland. She had no petechiae, ecchymosis, bruising, organomegaly, or lymphadenopathy.

Initial laboratory studies showed anemia with hemoglobin of 7.9 g/dl (normal range, 12–16 g/dl), thrombocytopenia with platelets of 4000/μl (140,000-450,000/μl), elevated lactate dehydrogenase of 653 IU/L (91–200 IU/L), haptoglobin < 6 mg/dl (30–225 mg/dl), reticulocyte count 5.31% (0.4–2.5%), high D-dimer of 1725 ng/ml (< 501 ng/ml), high fibrinogen of 633 mg/dl (232–519 mg/dl), and acute renal insufficiency with creatinine 1.20 mg/dl (0.44–1.0 mg/dl) (Table 1). Her peripheral smear showed many schistocytes. The result of her direct Coombs test was negative. Urinalysis showed moderate blood with 8-10 Red blood cells/high power field (0-2 RBCs/hpf) and 100 mg/dl proteins. Her coagulation panel showed prothrombin time and activated partial thromboplastin time of 1.07 and 29 seconds, respectively.

**Table 1:** Initial laboratory results of the patient

Laboratory test	Result	Reference range
Hemoglobin	7.9 g/dl	12–16 g/dl
Platelets	4000/μl	140,000–450,000/μl
LDH	653 IU/L	91–200 IU/L
Haptoglobin	< 6 mg/dl	30–225 mg/dl
Reticulocyte count	5.31%	0.4–2.5%
D-dimer	1725 ng/ml	< 501 ng/ml
Fibrinogen	633 mg/dl	232–519 mg/dl
Creatinine	1.20 mg/dl	0.44–1.0 mg/dl
ADAMTS13	< 5%	> 61%
TSH	0.528 IU/ml	0.3–4.5 mIU/ml
Free T4	3.93 ng/dl	0.5–1.26 ng/dl
Total T4	21.23 μg/dl	5.28–9.27 μg/dl
Free T3	7.0 pg/ml	2.28–3.96 pg/ml
INR	1.07	0.88–1.15
aPTT	29	26–39

*Abbreviations: aPTT* activated partial thromboplastin time, *INR* international normalized ratio, *LDH* lactate dehydrogenase, *TSH* thyroid-stimulating hormone

Clinically, the diagnosis of TTP was made, and the patient was transferred to the intensive care unit for close monitoring. She was started on plasmapheresis and intravenous steroids as part of the treatment for TTP. Her ADAMTS13 taken on admission came back a few days later as < 5% (normal, > 61%). Workup for secondary causes of TTP was also done. Her antinuclear antibody was positive at 7.01 (0–0.9), but results of other serological workup, including human immunodeficiency virus, hepatitis profile, anti-double-stranded deoxyribonucleic acid (DNA), cardiolipin antibodies, lupus screen, scleroderma antibodies, and QuantiFERON test (Qiagen, Germantown, MD, USA), were negative. She had positive centromere antibodies, but clinically she had no signs of CREST syndrome (calcinosis cutis, Raynaud phenomenon, esophageal dysfunction, sclerodactyly, and telangiectasia) or scleroderma. Her complement levels of C3 and C4 were found to be normal.

On further query, the patient reported that her mother had had Graves disease and her twin sister also had had Graves disease and had died of cardiac arrest at the age of 24 in the setting of thyrotoxicosis. The patient’s outpatient records 4 months prior to admission showed a free T4 of 7.7 ng/dl (0.5–1.26 ng/dl) and a thyroid-stimulating hormone (TSH) < 0.005 mIU/ml (0.3–4.5 mIU/ml). Outpatient ultrasound showed an enlarged thyroid with increased background vascularity. The patient’s platelet count was 248,000/μl (140,000–450,000/μl) at that time. She was started on methimazole and was advised to follow up with an endocrinologist, but she did not comply. No autoantibody levels were measured at that time, and she did not have an uptake scan.

On admission, her TSH was found to be 0.528 IU/ml (0.3–4.5 mIU/ml), free T4 was 3.93 ng/dl (0.5–1.26 ng/dl), total T4 was 21.23 μg/dl (5.28–9.27 μg/dl), and free T3 was 7.0 pg/ml (2.28–3.96 pg/ml). Her TSH receptor antibody and thyroid-stimulating immunoglobulin were found to be normal with a positive anti-thyroid peroxidase antibody. Unfortunately, these were drawn following several sessions of plasmapheresis; therefore, the absence or presence of thyroid autoimmune antibodies was not thought to be reliable for diagnosis of autoimmune thyroid disease. The patient’s Graves disease diagnosis was based on thyromegaly, glandular hypervascularity, and lack of biochemical improvement despite over 4 months of thionamide therapy. In preparation for thyroidectomy, she was treated with propylthiouracil, potassium iodide oral solution, cholestyramine, prednisone, and propranolol. Her thyroid pathology revealed a diffuse thyrotoxic goiter with patchy chronic inflammation.

She was given multiple sessions of plasmapheresis, along with a high dose of steroids, cyclophosphamide, and rituximab. She initially showed marked improvement in her platelet count; however, her hospital course was complicated by hospital-acquired pneumonia possibly provoked by severe immunodeficiency, sepsis, and acute respiratory distress syndrome (ARDS) with ventilator-dependent respiratory failure requiring rotoproning, and her course was further complicated by hemothorax requiring chest tube placement. All these complications led to a subsequent decline in her platelet count. A new medication recently approved for acquired TTP called caplacizumab, a monoclonal antibody to vWF, was considered in her management. Her platelets stabilized at approximately 50,000/μl (140,000–450,000/μl), and her ADAMTS13 activity normalized, so the caplacizumab was not administered. However, due to overwhelming ARDS, her hypoxemia drove her heart into pulseless electrical activity. Despite multiple rounds of cardiopulmonary resuscitation, she died.

## Discussion

TTP is a rare disease with an incidence of approximately 3 cases per 1 million adults per year [3]. TTP usually involves microangiopathic hemolytic anemia, thrombocytopenia, and renal injury [3]. ADAMTS13 is a metalloproteinase that breaks down vWF; when it is deficient, large multimers of vWF circulate, provoke the formation of thrombi, and cause widespread platelet activation leading to platelets’ subsequent deficiency and destruction [3]. It is associated with other autoimmune diseases, such as ITP, Sjögren syndrome, and systemic sclerosis; however, it is not commonly associated with Graves disease. It has inherited and acquired forms [4]. The mortality associated with TTP can be as high as 90%, and clinical diagnosis is the key to rapid management [4].

Treatment of TTP depends on the risk stratification of patients based on clinical and laboratory data present at the time of diagnosis. Several therapies are available, including plasma exchange (PEX), high-dose steroids, rituximab, and caplacizumab. Daily PEX is the mainstay of treatment. It allows removal of autoantibodies and repletes ADAMTS13. If there is any delay in PEX, then fresh frozen plasma transfusion should be considered. Platelet transfusion is contraindicated unless bleeding is life-threatening [5]. Caplacizumab is a humanized monoclonal antibody fragment that binds to vWF and blocks vWF interaction with platelet glycoprotein lb-IX-V. As indicated by faster platelet count normalization, it prevents further consumption of platelets into microthrombi and the consequent progression of tissue ischemia [6].

The overall prevalence of hyperthyroidism in the United States is 1.2%, with an incidence of 20 per 100,000 to 50 per 100,000 population. Graves disease constitutes the majority of hyperthyroid cases [7]. It is also more common in females and between ages 20 and 50 years [7]; our patient fell within these parameters. Patients on antithyroid medications must be monitored frequently for liver toxicity and agranulocytosis, which are rare adverse effects of thionamide therapy. The use of methimazole prior to admission was not thought to be a cause of our patient’s blood cell abnormalities, because her white blood cell count was normal on admission. Drug-induced thrombotic microangiopathy (DITMA) was also ruled out because methimazole has not been reported to cause DITMA. Our patient received antithyroid medications, as well as adjunct medications (cholestyramine, steroids, and potassium iodide drop) to bridge to total thyroidectomy. Radioactive iodine ablation was not possible while she was an inpatient, because she still required daily plasmapheresis, and quarantine following ablation could not be assured.

In our review of the literature, we found that only a few cases of TTP co-occurring with Graves disease have been described. In their case report, Chhabra *et al.* mentioned one woman who developed sustained remission of TTP after treatment of her hyperthyroidism with radioactive iodine [8]. Our patient began experiencing symptoms of TTP a few days after stopping methimazole, and her T4 and T3 were very elevated on admission, leading us to consider whether her uncontrolled hyperthyroidism could have provoked her TTP. In their case report, Bellante *et al.* mentioned that their patient showed improvement in TTP with only radioactive iodine therapy and methylprednisolone. PEX was avoided, and there was no relapse of TTP in the 6-month follow-up period [9]. The question has been raised before whether treating the hyperthyroid state could concurrently control TTP [10].

Because both Graves disease and TTP are autoimmune diseases, Lhotta *et al*. hypothesized that Graves disease triggers some autoimmune processes leading to the production of antibodies to ADAMTS13 [11]. Another report illustrated two cases of Graves disease possibly causing a relapse of preexisting TTP; with successful treatment of both TTP and Graves disease, there was rapid and prolonged remission of Graves disease [11].

## Conclusion

The association between Graves disease and TTP seems to be more than coincidental. Graves disease patients receiving thionamide therapy should be monitored for agranulocytosis by checking the complete blood count. If there are any abnormalities in the cell count, especially thrombocytopenia, clinicians should consider the possibility of another autoimmune process, such as TTP, in the appropriate clinical context. Emphasis should be placed on controlling hyperthyroidism and avoiding lapses in medical therapy to prevent complications, as in our patient’s case.

TTP and Graves disease have been described infrequently in the literature. We present a case of TTP occurring in a patient with inadequately controlled Graves disease. This indicates that there may be a causal link. TTP is an important association to be aware of in patients with Graves disease and highlights the importance of adequate control of Graves disease.

## Data Availability

Data sharing is not applicable to this article, because no datasets were generated or analyzed during the current study.
